# Account of a Successful Operation for a Congenital Division of the Velum of the Palate and the Uvula

**Published:** 1820-03

**Authors:** M. Roux

**Affiliations:** Chirurgien-en-chef adjoint de l'Hopital de la Charité.


FOR THE LONDON MEDICAL AND PHYSICAL JOURNAL.

***Account of a successful Operation for a Congenital Division of the Velum of the Palate and the Uvula.*** **By M. Roux, Chirurgien-en-chef adjoint de l'Hopital de la Charité.**

THE patient was a young man, in whom the velum of the palate and the uvula were completely divided, as a congenital malformation: the voice was altered, as it commonly is in similar cases. The two edges of the division could be easily approached to each other; the mouth was large; the pharynx not very sensible; and the patient earnestly desired to have something done for his relief. Every thing concurred to favour the attempt of the operation.

M. Roux first passed three ligatures of waxed thread through the parts to be united, by means of a curved needle mounted on a handle: this prevented the too great retraction of the parts, from the irritation produced by the instrument in removing the edges of the velum and uvula, which was done to the extent of about half a line on each line: the ligatures were then tied, and the threads cut close to the knots.

Immediately after the operation, the voice became like what it is in the ordinary state. The patient was ordered to abstain wholly from food for three days, and to preserve perfect silence. In a few days the union was complete; but the two margins of the uvula are not perfectly correspondent; one of them descends a little lower than the other; and M. Roux intends to remove the projecting part by excision.

